# TET1-mediated DNA hypomethylation regulates the expression of MUC4 in lung cancer

**DOI:** 10.18632/genesandcancer.139

**Published:** 2017-03

**Authors:** Seiya Yokoyama, Michiyo Higashi, Hideaki Tsutsumida, Jouji Wakimoto, Tomofumi Hamada, Edwin Wiest, Kei Matsuo, Ikumi Kitazono, Yuko Goto, Xin Guo, Taiji Hamada, Sohsuke Yamada, Tsubasa Hiraki, Suguru Yonezawa, Surinder K. Batra, Michael A. Hollingsworth, Akihide Tanimoto

**Affiliations:** ^1^ Department of Pathology, Research Field in Medicine and Health Sciences, Medical and Dental Sciences Area, Research and Education Assembly, Kagoshima University, Sakuragoaka, Japan; ^2^ Center for the Research of Advanced Diagnosis and Therapy of Cancer, Graduate School of Medical and Dental Sciences, Kagoshima University, Japan; ^3^ Eppley Institute for Research in Cancer, Fred and Pamela Buffet Cancer Center, University of Nebraska Medical Center, NE, USA; ^4^ Department of Respiratory Medicine, Minami-kyushu National Hospital, Aira, Japan; ^5^ Department of Oral Surgery, Kagoshima University Medical and Dental Hospital, Sakuragoaka, Japan; ^6^ Department of Biochemistry and Molecular Biology, Eppley Institute for Research in Cancer and Allied Diseases, University of Nebraska Medical Center, NE, USA

**Keywords:** Lung cancer, pathology, DNA methylation, risk factor, MUC4

## Abstract

Lung cancer remains a disease of high mortality, despite advanced diagnostic techniques. Mucins (MUC) play crucial roles in carcinogenesis and tumor invasion in lung neoplasms. Our immunohistochemistry (IHC) studies have shown that high MUC4 expression correlates with a poor outcome. We have also shown that the expression of several mucin genes in cancer cell lines is regulated by DNA methylation. We evaluated the expression level of MUC4, mRNA and several DNA hypomethylation factors in lung tissue samples from 33 patients with various lung lesions. The results indicated that the DNA methylation status of *MUC4* matched the expression level of mRNA. In addition, the *TET1* (Ten-Eleven Translocation) mRNA showed a significant correlation with the status of DNA methylation of *MUC4*. Furthermore, the treatment of a lung cancer cell line with *TET1* siRNA caused a reduction in *MUC4* mRNA expression. Thus, we suggest that *TET1* mediated DNA hypomethylation plays a key role in the expression of MUC4. This is the first report that TET1 mediated DNA hypomethylation regulates the expression of MUC4 in lung cancer. The analysis of these epigenetic changes may be useful for diagnosing carcinogenic risk.

## INTRODUCTION

Lung cancer is the leading cause of cancer-related death in most industrialized countries [1, 2], and adenocarcinomas account for approximately 45% of lung cancer [3]. Poor prognosis for patients with lung cancer has persisted, despite efforts in primary prevention, screening and therapy [1]. Within current screening techniques for patients that lack symptoms, a diagnostic method to differentiate small lung adenocarcinomas from benign lesions is needed.

Mucins (MUC) play crucial roles in carcinogenesis and tumor invasion in lung neoplasms. MUC4, a large membrane-bound glycoprotein that is translated as a single polypeptide, undergoes intracellular autocatalytic proteolytic cleavage into two subunits that form a stable non-covalent heterodimer that is transported to the cell surface. MUC4 plays an important role in cell proliferation and differentiation of epithelial cells by inducing specific phosphorylation of ErbB2 and enhancing the expression of the cyclin dependent kinase inhibitor p27, which inhibits cell cycle progression [4–11]. Our immunohistochemistry (IHC) studies have revealed that a high MUC1/SP-A ratio is strongly associated with a poor outcome in patients with small-size lung adenocarcinoma and that high MUC4 expression in lung adenocarcinoma patients associates with poor outcome [12–14]. We have also found that the methylation status, mRNA expression, and protein expression of mucins in cancer cell lines are correlated [15–17]. We have shown that mucin gene expression is regulated by DNA methylation status in pancreatic tissue [18, 19]. In addition, we reported that hypomethylation of the *MUC4* promoter correlates with a decreased overall survival in pancreatic ductal adenocarcinoma [20].

Bisulfite treatment is a current standard for DNA methylation analysis. However, one pitfall with the bisulfite treatment is that 5-hydroxy methyl cytosine (5hmC) is detected as 5-methyl cytosine (5mC). 5hmC is the primary product of 5mC oxidation, a process that plays an essential role in normal embryonic development and the maintenance of pluripotency and stem cell reprogramming [21–24]. Recently, it was reported that not only DNA methylated by the Dnmt (DNA methyltransferase) family but also DNA modified by TET (Ten-Eleven Translocation) family, AICDA (activation-induced deaminase)/APOBEC (apolipoprotein B mRNA editing enzyme, catalytic polypeptide) family and/or GCM1 (glial cells missing 1) convert 5mC to 5hmC and higher oxidation products in mammalian genomes (i.e. active DNA hypomethylation) [25–31].

In this study, we sought to further characterize the epigenetic changes of the *MUC4* promoter region in lung adenocarcinomas through analysis of DNA samples with the MSE method (with bisulfite treatment and/or TET assisted bisulfite treatment). As no recent study has evaluated the extent of 5hmC modification of the *MUC4* gene and correlated this to expression levels of *MUC4* mRNA in lung tumors, we analyzed *MUC4* 5mC status and/or 5hmC status in lung tissue to study the relationship between *MUC4* promoter modification and expression.

## RESULTS

### Correlation between DNA methylation status and mRNA expression

**Figure 1:**
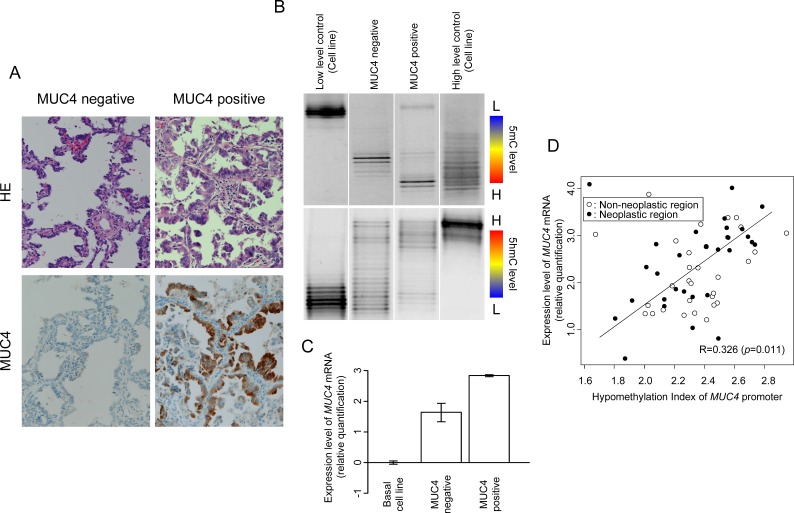
Analysis of MUC4 expression and methylation status in human lung samples (A) Expression of MUC4 protein examined by immunohistochemical staining. HE: Hematoxylin and Eosin Staining. Magnification: ×200. (B) DNA methylation status (upper) and hydroxy methylation status (lower) of the *MUC4* promoter region. The MSE detected these statuses using bisulfite treatment or TAB treatment of the DNA. L: Low methylation (or hydroxy methylation). H: High methylation (or hydroxy methylation). MUC4 negative tissue showed high methylation and low hydroxy methylation. MUC4 positive tissue showed high methylation and high hydroxy methylation. (C) Expression of *MUC4* mRNA examined by quantitative real time RT-PCR. The bar graphs show gene expression levels relative to those in A427 cells. (D) Multiple regression analysis of *MUC4* mRNA expression against DNA hypomethylation status in *MUC4* promoter, calculated by 5mC and 5hmC score. R: correlation coefficient, ○: non-neoplastic region, ●: neoplastic region.

In total, 66 lung tissue samples were collected from 33 lung cancer patients (Table S1). We examined the relationship between *MUC4* mRNA expression, DNA methylation of the promoter, and IHC staining for MUC4 protein in paired lung tissues. Representative cases of mRNA expression (RT-PCR) paired with IHC analysis and 5mC score and 5hmC score are shown in Figure 1. We found that IHC positive samples were mRNA positive and that IHC negative samples were mRNA negative (Figure 1A and 1C). We observed similar methylation patterns of 5mC in both MUC4 positive and MUC4 negative lung tissues; however, 5hmC status was correlated with expression of MUC4 protein (Figure 1B). We analyzed the relationship between the 5mC or the 5hmC score of the *MUC4* promoter and the expression level of *MUC4* mRNA with Pearson's correlation coefficient (R=-0.323, *p*=0.011 and R=0.105, *p*=0.426, Table 1). A significant degree of correlation was observed between the hypomethylation index (calculated by the following formula: hypomethylation index = 2.94+(1.32(5hmC score)-0.98(5mC score))/1000) and mRNA expression of *MUC4* (R=0.326, *p*=0.001, Figure 1D).

**Table 1:** Summarized correlation coefficient (R)

	Expression level of mRNA							
	*MUC4*	*TET1*	*TET2*	*TET3*	*Dnmt1*	*Dnmt3a*	*GCM1*	*AICDA*
Expression level of mRNA								
*MUC4*	NA	0.543	−0.111	0.144	0.420	0.523	0.458	0.392
Methylation status in MUC4 promoter								
5hmC score	0.105	0.319	−0.055	0.029	0.161	0.308	0.198	0.114
5mC score	−0.323	−0.364	−0.404	−0.283	−0.551	−0.619	−0.338	−0.383
Hypomethylation Index	0.326	0.392	0.388	0.280	0.551	0.636	0.352	0.385

### Differences in methylation status between neoplastic and non-neoplastic areas

**Figure 2:**
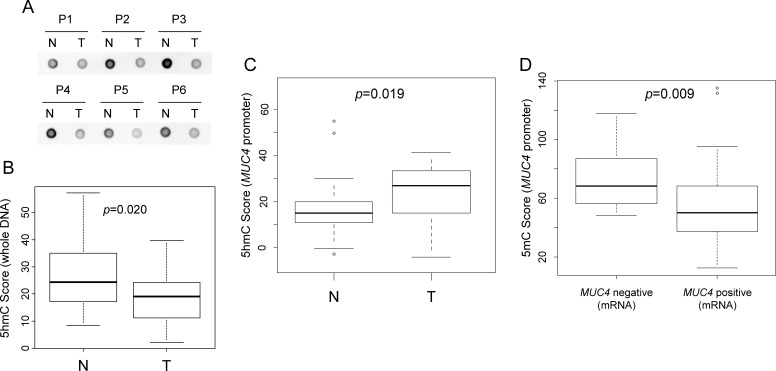
Comparison of 5mC and 5hmC scores between neoplastic and non-neoplastic regions (A) Dot blot analysis of 5hmC in whole DNA. These patients (P1 to P6) showed higher 5hmC signal in the non-neoplastic region than in the neoplastic region. (B) Comparison of 5hmC scores in whole DNA between neoplastic and non-neoplastic regions. The 5hmC score in whole DNA was calculated by dot blot intensity in each sample and was normalized to the amount of DNA applied to the membrane. (C) Comparison of 5hmC scores in the *MUC4* promoter between neoplastic and non-neoplastic regions. (D) Comparison of 5mC scores in the *MUC4* promoter between *MUC4* negative and *MUC4* positive (mRNA analysis) samples. N: non-neoplastic region, T: neoplastic region.

Thirty-three neoplastic samples and 33 paired non-neoplastic samples were analyzed. No significant difference was observed for expression of *MUC4* mRNA in neoplastic tissues versus non-neoplastic tissues. However, there was a statistically significant difference in *MUC4* mRNA expression in samples positive or negative for MUC4 (as determined by IHC analysis) (*p*=0.013, Supplemental Figure 1A). A threshold value of *MUC4* mRNA expression that could distinguish between positive and negative MUC4 IHC staining was determined to be 2.127 by ROC analysis (Supplemental Figure 1B). A dot-blot analysis was used to examine differences in 5hmC modification of the *MUC4* gene between neoplastic and non-neoplastic regions obtained from lung tissues (Figure 2A). Non-neoplastic areas showed a significantly higher level of 5hmC than neoplastic areas (*p*=0.020, Figure 2B). On the other hand, 5hmC modification of the *MUC4* promoter region in non-neoplastic regions was lower than in neoplastic regions (*P*=0.019, Figure 2C). There was no significant difference in 5hmC modification of the *MUC4* promoter region between neoplastic and non-neoplastic regions. However, within the *MUC4* mRNA negative group, higher levels of 5mC modification were observed compared to that of the *MUC4* mRNA positive group (*p*=0.009, Figure 2D). These data are summarized in Table 2. These results suggest that, including 5hmC, the neoplastic area has an increased hypomethylation status in the *MUC4* promoter region. However, overall 5hmC modification within the neoplastic areas was lower than in the non-neoplastic areas.

Table 2Comparison of expression level and methylation status

**Table:** 

a. between non-neoplastic and neoplastic region								
	Non-neoplastic region			Neoplastic region				
	n	mean	±sd	n	mean	±sd	*p* value	
Methylation status of *MUC4* promoter								
5mC	(31)	63.82	±25.01	(31)	65.18	±29.64	0.846	
5hmC	(33)	18.64	±17.65	(33)	31.77	±33.05	0.019	*
Expression level of mRNA								
MUC4	(33)	2.20	±0.75	(33)	2.44	±0.89	0.226	
*TET1*	(33)	1.85	±0.44	(33)	1.92	±0.63	0.602	
*TET2*	(31)	0.27	±0.70	(31)	0.29	±0.57	0.910	
*TET3*	(32)	2.60	±0.72	(32)	2.56	±0.79	0.867	
*AICDA*	(33)	2.34	±0.55	(33)	2.30	±0.64	0.792	
*GCM1*	(33)	1.65	±0.42	(33)	1.61	±0.65	0.730	**
*Dnmt1*	(32)	−0.02	±0.53	(32)	0.03	±0.58	0.742	
*Dnmt3a*	(32)	0.05	±0.39	(32)	0.04	±0.47	0.872	

*Willcoxon T test, **unequal T test.

**Table:** 

b. between expression level of MUC4 mRNA positive and negative								
	positive			negative				
	n	mean	±sd	n	mean	±sd	*p* value	
Methylation status of *MUC4* promoter								
5mC	(33)	56.99	±29.58	(27)	74.67	±20.77	0.009	
5hmC	(36)	30.02	±34.61	(30)	19.43	±11.75	0.446	*
Expression level of mRNA								
*TET1*	(36)	2.08	±0.60	(30)	1.65	±0.35	0.001	**
*TET2*	(32)	0.26	±0.63	(30)	0.29	±0.65	0.864	
*TET3*	(34)	2.73	±0.61	(30)	2.41	±0.86	0.098	
*AICDA*	(36)	2.42	±0.57	(30)	2.21	±0.60	0.165	
*GCM1*	(36)	1.76	±0.56	(30)	1.47	±0.48	0.027	
*Dnmt1*	(34)	0.14	±0.52	(30)	−0.16	±0.56	0.029	
*Dnmt3a*	(34)	0.19	±0.41	(30)	−0.11	±0.40	0.004	

Threshold value of positive MUC4 expression is >2.127, *Willcoxon T test, **unequal T test.

**Table:** 

c. between methylation status of MUC4 promoter								
	hypomethylation			hypermethylation				
	n	mean	±sd	n	mean	±sd	*p* value	
Expression level of mRNA								
*TET1*	(18)	2.21	±0.68	(42)	1.69	±0.38	0.006	**
TET2	(15)	0.43	±0.75	(41)	0.23	±0.62	0.350	
*TET3*	(16)	2.98	±0.53	(42)	2.48	±0.78	0.008	
*AICDA*	(18)	2.58	±0.56	(42)	2.14	±0.55	0.008	
*GCM1*	(18)	1.93	±0.52	(42)	1.46	±0.49	0.003	
*Dnmt1*	(16)	0.43	±0.29	(42)	−0.22	±0.53	<0.001	**
*Dnmt3a*	(16)	0.4	±0.24	(42)	−0.17	±0.35	<0.001	

Threshold value of hypomethylation index is >2.489, **unequal T test

### 5mC/5hmC score and expression of DNA methylation-related enzymes in lung tissue

The mRNA expression levels of DNA methylation-related enzymes (*DNMT1* and *DNMT3a*) and DNA demethylation-related enzymes (*TET1*, *TET2*, *TET3*, *AICDA* and *GCM1*) in neoplastic and non-neoplastic samples are summarized in Table 2. There were no differences in expression of these between neoplastic and non-neoplastic regions. However, a comparison between the *MUC4* mRNA positive group and negative group revealed significant differences in expression levels of *TET1*, *GCM1*, *Dnmt1* and *Dnmt3a* (*p*=0.001, *p*=0.027, *p*=0.029 and *p*=0.004, respectively). The expression level of *TET1* showed a significant correlation with the expression level of *MUC4* (R=0.543, p<0.001, Table 1). To examine whether the *MUC4* promoter hypomethylation is influenced by the expression of DNA methylation-related enzymes, we analyzed the expression level of these enzymes in the hypomethylated and hypermethylated groups. The threshold value of methylation index to distinguish between hypomethylation and hypermethylation of the *MUC4* promoter was 2.489 as determined by ROC analysis (Supplemental Figure 1C and 1D). The hypomethylated group showed higher expression levels of *TET1*, *TET3*, *GCM1*, *AICDA*, *Dnmt1* and *Dnmt3a* than the hypermethylated group (Table 2). The expression level of *TET1*, *TET2*, *AICDA*, *Dnmt1* or *Dnmt3a* correlated with the hypomethylation index (R=0.392, R=0.388, R=0.385, R=0.551 and R=0.636, respectively, Table 1). In order to find statistically significant differences between enzymes related to DNA methylation, we performed a multiple regression analysis. We determined the best regression formula with the least variables (five DNA demethylation-related enzymes) with the lowest AIC values for the hypomethylation status of *MUC4* as follows: Fm (Enzyme expression index for *MUC4*) = 1.8 + 0.23(*TET1*) + 0.17(*TET2*). This predictive model showed a significantly high correlation with the hypomethylation index of *MUC4* (R2 = 0.562, p<0.001, Supplemental Figure 2).

### Correlation between expression level of DNA methylation-related enzymes and hypomethylation status of MUC4 and clinicopathological features

**Figure 3:**
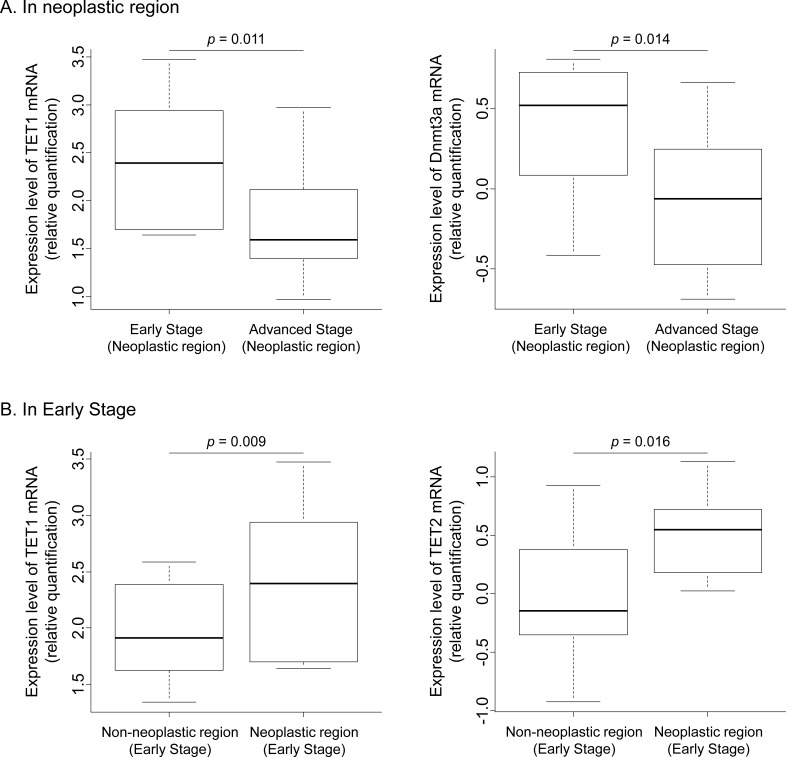
Expression analysis of *TET1*, *TET2*, and *Dnmt3a* mRNA (A) Comparison between early and advanced stage in the neoplastic region. (B) Comparison between the non-neoplastic and neoplastic regions in early stage. These mRNA expression levels were evaluated by relative quantification.

Expression levels of DNA methyltransferases (DNMTs) as DNA methylation factors (*DNMT1* and *DNMT3a*), DNA demethylation factors (*TET1*, *TET2*, *TET3*, *AICDA* and *GCM1*) and *MUC4* were evaluated in tumors representative of early stage (Tumor size < 10mm), later stages, lymphatic permeation negative and positive samples, and vascular permeation negative and positive samples (summarized in Table S3). Analysis of vascular permeation negative and positive samples revealed no significant differences in DNA methylation-related enzymes, *MUC4* expression levels, or *MUC4* methylation status. However, analysis of samples of the neoplastic region without lymphatic permeation showed higher expression of *TET1*, *Dnmt1,* and *Dnmt3a* than samples of the neoplastic region with lymphatic permeation (*p*=0.020, *p*=0.032 and *p*=0.005 respectively). In the case of samples with lymphatic permeation, the neoplastic region showed a higher 5hmC score in the *MUC4* promoter than the paired non-neoplastic region (*p*=0.004). Early stage lung cancers showed higher expression of *TET1* and *Dnmt3a* than other advanced stages (*p*=0.011 and *p*=0.014 respectively, Figure 3A). In early stage samples, the neoplastic region showed higher *TET1* and *TET2* expression than the paired non-neoplastic region (*p*=0.009 and *p*=0.016 respectively, Figure 3B).

### Effect of TET1 knockdown on MUC4 expression in cancer cell lines

**Figure 4:**
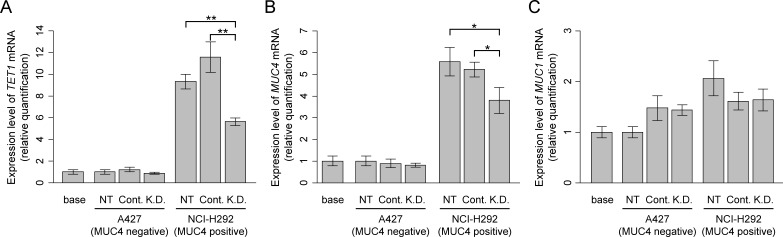
Effect of *TET1* siRNA treatment (A) Expression analysis of *TET1* mRNA. NCI-H292 has a high expression level of *TET1* mRNA and showed significant decrease in *TET1* expression after *TET1* siRNA treatment. (B) Expression analysis of *MUC4* mRNA. NCI-H292 has a high expression level of *MUC4* mRNA and showed a significant decrease in the expression of *MUC4* after *TET1* siRNA treatment. (C) Expression anaylsis of *MUC1* mRNA. Both NCI-H292 and A427 show no significant difference in *MUC1* mRNA expression upon *TET1* siRNA treatment. NT: non-treated, Cont.: control siRNA treatment, K.D.: *TET1* siRNA treatment. The bar graphs show gene expression levels relative to those in the non-treated A247

To further explore a causal relationship between *TET1* expression and activity and *MUC4* expression, lung cancer cell lines (A427 and NCI-H292) were employed. When endogenous *TET1* in A427 cells (MUC4 positive) was knocked down by siRNA (Figure 4A), the *MUC4* expression level was strongly reduced (*p*=0.001). In contrast, siRNA knockdown of *TET1* in the *MUC4* negative NCI-H292 cell line was ineffective in changing *MUC4* expression (Figure 4B). Also, knockdown of *TET1* caused no change in *MUC1* expression (Figure 4C). These data suggest that TET1 plays a key role in regulating the expression of *MUC4* mRNA.

## DISCUSSION

In the present study, we analyzed the correlation between *MUC4* expression and DNA methylation and 5mC and/or 5hmC scores in the promoter region of *MUC4* in lung adenocarcinomas. It has been shown previously that expression of mucin genes such as *MUC1*, *MUC2*, *MUC3*, *MUC4,* and *MUC5AC* are regulated by DNA methylation (5mC) of these promoter regions [15–17]. Our results are the first to demonstrate that TET1-mediated DNA hypomethylation regulates the expression of *MUC4* in lung adenocarcinomas.

In our comparison of lung neoplastic and non-neoplastic samples, we found a significant difference in 5hmC scores in the *MUC4* promoter region. However, the level of 5mC in the promoter of *MUC4* showed no difference when the neoplastic non-neoplastic regions were compared, while the 5hmC score of the *MUC4* promoter was increased in the neoplastic region when compared with the non-neoplastic region. In contrast, in whole DNA, the non-neoplastic region of the lung showed higher 5hmC scores when compared with the neoplastic region. Some recent studies have also shown that 5hmC is substantially decreased in human prostate, breast, colon, lung, liver, and pancreatic cancers, as well as glioma and melanoma [24, 32–36]. Therefore, our results show that alteration of the DNA hypomethylation process, such as that of the *MUC4* promoter region, can be a gene-specific process that persists in spite of overall trends in hypomethylation. The expression level of *MUC4* was not found to be different when comparing the neoplastic region to the non-neoplastic region. This may be because MUC4 is expressed only in neoplasms with poor outcome, as most lung tissue does not express MUC4. Thus, these results suggest that the DNA hypomethylation process, conversion of 5mC to 5hmC in the *MUC4* promoter, precedes increases in expression of MUC4 in the lung neoplastic region.

Our evaluation of the relationship between *MUC4* expression level and the degree of hypomethylation of *MUC4* revealed that the group with high expression of *MUC4* mRNA showed a higher 5mC score of *MUC4* than the group with low expression of *MUC4* mRNA. The hypomethylation score (calculated by comparison of 5mC and 5hmC scores) showed a significant correlation with the expression of *MUC4* mRNA. These results complement the results in lung cancer cell lines that we found in our previous study [16] and suggest that MUC4 expression is regulated by epigenetic DNA modification (e.g., 5mC and/or 5hmC) in lung adenocarcinomas as well as in non-neoplastic lung tissue.

Concerning the relationship between *MUC4* promoter hypomethylation and expression of several epigenetic alteration factors such as the TET family, AICDA/Apobec family, GCM1 and Dnmt family, we found significant differences between the *MUC4* hypomethylated group and the *MUC4* hypermethylated group. The expression levels of the active hypomethylation factors *TET1*, *TET3*, *GCM1* and *AICDA* in the *MUC4* hypomethylation group were significantly higher than in the *MUC4* hypermethylation group. Similarly, the *MUC4* hypomethylation group showed a significantly higher expression level of DNA methylation factors, *Dnmt1* and *Dnmt3a*, than the *MUC4* hypermethylation group. Multiple correlation analysis showed that the expression levels of *TET1* and *TET2* significantly correlated with the hypomethylation index of the *MUC4* promoter. These results suggest that expression of the *MUC4* gene is increased when 5mC levels and/or 5hmC modifications at the *MUC4* promoter region are altered, and this alteration may be caused by activation of these DNA methylation-related enzymes.

A comparison of DNA methylation-related enzymes, *MUC4* methylation status and clinicopathological information, revealed significantly higher expression of *TET1*, *Dnmt1* and *Dnmt3a* in the neoplastic region with lymphatic permeation than in the neoplastic region without it. Interestingly, in our samples *TET1* was downmodulated according to the tumor size. Moreover, *TET1* expression was significantly higher in the early stage (tumor size < 10mm) neoplastic region than in the paired non-neoplastic region. This result suggests that *TET1* expression is the initial step in reprogramming DNA methylation in lung cancer. In addition, we found a significant correlation between *TET1* expression and *MUC4* mRNA expression. We showed a significant reduction of *MUC4* mRNA by *TET1* mRNA down-regulation in a lung cancer cell line. We suggest that increased expression of TET1 may cause hypomethylation and/or the conversion of 5mC to 5hmC or higher oxidation products in the *MUC4* promoter region. These results suggest that demethylation of the *MUC4* promoter by TET1 may be involved in the early stage and/or in the production of precursor cancer cells of lung cancer.

In summary, our data demonstrate that *MUC4* expression is increased by DNA hypomethylation when both 5mC and 5hmC are considered. Furthermore, *MUC4* hypomethylation status is statistically associated with active methylation and/or hypomethylation factors. Moreover, in the early stage, TET1 plays a key role in *MUC4* hypomethylation. Thus, detection of the hypomethylation index of *MUC4* and these DNA methylation-related factors has potential clinical value as an indicator of overall survival and should be evaluated further for clinical utility. Since MUC4 is a key mucin in pathological diagnosis of lung neoplasms [12, 14], our goal is to apply DNA methylation analysis of this gene using bronchoalveolar lavage fluid and/or sputum for early diagnosis of lung neoplasms.

## MATERIALS AND METHODS

### Cell lines

Human lung carcinoma cell lines A427 and NCI-H292 were obtained from the American Type Culture Collection. A427 was cultured in Eagle's minimum essential medium (Sigma, St. Louis, MO, USA), and NCI-H292 was cultured in RPMI 1640 medium (Sigma, St. Louis, MO, USA). The media was supplemented with 10% fetal bovine serum (Invitrogen, Minatoku, Tokyo, Japan) and 100 U/mL of penicillin and 100 μg/mL streptomycin (Sigma).

### Clinical samples

#### Lung tissue samples

We aimed to examine the relationship between the extent of DNA methylation of mucin genes and expression of mRNA in paired lung tissues. We obtained tissue blocks (about 2×2×2 mm) with neoplastic and non-neoplastic areas from surgically resected fresh specimens of 30 adenocarcinomas, 2 squamous cell carcinomas, and 1 adenosquamous carcinoma and paired non-neoplastic samples. Table S1 summarizes the clinicopathological characteristics of the samples analyzed herein.

#### Ethics statement

The study was conducted in accordance with the guiding principles of the Declaration of Helsinki. Collection of samples was approved by the ethical committees of the hospital and informed written consent was obtained from each patient. All studies using human materials in this article were approved by the Ethical Committee of Kagoshima University Hospital (revised 20-82, revised 22-127, and revised 26-145).

#### Extraction and Quantification of mRNA

Total RNA was extracted from cell lines and human lung tissues using an RNeasy Mini kit (QIAGEN, Tokyo, Japan). Total RNA (1 μg) was reverse transcribed with a high capacity RNA-to-cDNA Kit (Applied Biosystems, CA, USA). Real-time reverse transcription–PCR was performed on a Roche LightCycler^®^ 96 System using FastStart Essential DNA Green Master (Roche, Tokyo, Japan). Gene expression was normalized to the β-actin mRNA level in each sample. The data of the A427 cell line were used for basal control. Primer sets are shown in Table S2.

#### Dot blot analysis

DNA was denatured in 0.4 M NaOH, 10 mM EDTA at 95°C for 10 minutes, and then neutralized by adding an equal volume of cold 2 M ammonium acetate (pH 7.0). Next, 2-fold dilutions of denatured DNA samples were spotted on a Hybond N+ nylon membrane. The DNA was fixed by UV cross-linking, washed with 2x SSC buffer and air-dried. The membrane was then blocked with 5% non-fat milk and incubated with polyclona 5hmC antibody (1:1000) (active motif). Binding of an HRP-conjugated secondary antibody (1:12,000) was visualized by enhanced chemiluminescence. The blot intensity was measured by Image J software (National Institutes of Health <http://rsb.info.nih.gov/ij/>). The dot blot intensity in each sample was normalized to the amount of DNA applied to the membrane.

#### Extraction of DNA and Bisulfite Modification

DNA from cell lines and lung tissue was extracted using a DNeasy Tissue System (QIAGEN). Bisulfite modification of the genomic DNA was carried out using an Epitect Bisulfite Kit (QIAGEN). Purification of PCR products was carried out using a Wizard SV Gel and PCR Clean-Up System (Promega).

#### TET1 assisted bisulfite (TAB) treatment

For measuring 5hmC in the *MUC4* promoter, collected total DNA was treated by TAB treatment similar to that used by Yu et al. [36]. In this method, to protect 5hmC, the DNA sample was treated with β-glucosyltransferase. Subsequently, recombinant TET1 was used to convert 5mC to 5-formylcytosine (5-fC) and/or 5-carboxylcytosine (5-caC). After the bisulfite treatment and PCR amplification, both cytosine (C) and higher oxidation products of 5mC (i.e. 5-fC and 5-caC) are converted to thymine (T), whereas protected 5hmC remains C.

#### MSE Analysis

MSE analysis was performed using previously described methods [19]. Briefly, the target DNA fragments were amplified by nested PCR using bisulfite treated DNA using the primer sets shown in Table S2. In the electrophoresis step, the amplicon was applied to the D-Code system (BioRad Laboratories, Hercules, CA, USA) using a polyacrylamide gel with a linear denaturant gradient at 60°C and 70 V for 14 h. Band intensity was quantified by Image J software. The 5mC score and 5hmC score were calculated as the proportion of highest band intensity to total band intensity of the sample. Subsequently, these scores in each sample were normalized using data from a hypomethylated and hypermethylated control. Cell lines that are hyper- and hypomethylated (Caco-2 and LS174T) were used as controls for determination of 5mC scores. An oligonucleotide sequence (all CpG hydroxy methylated version and an all CpG unmethylated version) was used as a control to detemermine 5hmC scores.

### Immunohistochemical Staining

Immunohistochemistry (IHC) was performed in cut sections of lung tumors using anti-MUC4 MAb clone 8G7 (MAb MUC4/8G7, the kind gift of Surinder K. Batra) [9] using the immunoperoxidase method. Antigen retrieval was performed using CC1 antigen retrieval buffer (pH 8.5, EDTA, 100°C, 30 minutes; Ventana Medical Systems, AZ, USA) for all sections. Following incubation in phosphate buffered saline, pH 7.4 (PBS) with 1% bovine serum albumin (BSA), sections were stained on a Benchmark XT automated slide stainer using a diaminobenzidine detection kit (UltraView DAB, Ventana Medical Systems). The control staining (normal mouse serum or PBS-BSA instead of the primary antibodies) showed no reaction.

### RNA interference

*TET1* knockdown was performed using MISSION^®^ esiRNA human *TET1* (Sigma-Aldrich, St. Louis, MO, USA) according to the manufacturer's instructions. MISSION^®^ siRNA Universal Negative Control (Sigma-Aldrich, St. Louis, MO, USA) was used as a control. Briefly, A427 and NCI-H292 cells were seeded in 6-cm dishes. At 50% confluency cells were transfected with 13.6 nmol/l siRNA using Lipofectamine RNAiMAX (Invitrogen, Carlsbad, CA, USA). After 48 h incubation, the cells were harvested.

### Statistical Analysis

Data were analyzed using the “R” computing environment [37]. The normality of the data distribution was evaluated by the Kolmogorov-Smirnov test. An F test was performed to compare the variances of the two samples from normal populations. A non-parametric test of two-group difference was performed by the Mann-Whitney U test. A parametric test of two-group difference was performed by the Welch t-test (Unequal variance) or Student t-test (Equal variance). A Bartlett test was performed to compare the variances of multiple samples from normal populations. A nonparametric test of multi-group difference was performed by the Kruskal-Wallis one-way analysis of variance. A parametric test of multi-group difference was performed by the one-way analysis of variance (ANOVA). The correlation coefficient (R) was determined by the Pearson product-moment correlation coefficient. The multiple regression analysis was performed with the general linear model and goodness of fit was analyzed with coefficient of determination (R squared) values. The threshold points were determined by ROC curve analysis. A *p*-value <0.05 was considered statistically significant.

## SUPPLEMENTARY MATERIALS FIGURES AND TABLES



## ABBREVIATIONS

MUC: Mucins
IHC: immunohistochemistry
TET: Ten-Eleven Translocation
5mC: 5-methyl cytosine
5hmC: 5-hydroxy methyl cytosine
Dnmt: DNA methyltransferase
AIDCA: activation-induced deaminase
APOBEC: apolipoprotein B mRNA editing enzyme, catalytic polypeptide
GCM: glial cells missing

## References

[1] Siegel R, DeSantis C, Virgo K, Stein K, Mariotto A, Smith T, Cooper D, Gansler T, Lerro C, Fedewa S, Lin C, Leach C, Cannady RS (2012). Cancer treatment and survivorship statistics, 2012. CA Cancer J Clin.

[2] Torre LA, Bray F, Siegel RL, Ferlay J, Lortet-Tieulent J, Jemal A (2015). Global cancer statistics, 2012. CA Cancer J Clin.

[3] Howlader N, Noone A, Krapcho M, Miller D, Bishop K, Altekruse S, Kosary C, Yu M, Ruhl J, Tatalovich Z, Mariotto A, Lewis D, Chen H (2016). SEER Cancer Statistics Review, 1975-2013.

[4] Chaturvedi P, Singh AP, Chakraborty S, Chauhan SC, Bafna S, Meza JL, Singh PK, Hollingsworth MA, Mehta PP, Batra SK (2008). MUC4 mucin interacts with and stabilizes the HER2 oncoprotein in human pancreatic cancer cells. Cancer Res.

[5] Chaturvedi P, Singh AP, Moniaux N, Senapati S, Chakraborty S, Meza JL, Batra SK (2007). MUC4 mucin potentiates pancreatic tumor cell proliferation, survival, and invasive properties and interferes with its interaction to extracellular matrix proteins. Mol Cancer Res.

[6] Jepson S, Komatsu M, Haq B, Arango ME, Huang D, Carraway CA, Carraway KL (2002). Muc4/sialomucin complex, the intramembrane ErbB2 ligand, induces specific phosphorylation of ErbB2 and enhances expression of p27(kip), but does not activate mitogen-activated kinase or protein kinaseB/Akt pathways. Oncogene.

[7] Jonckheere N, Perrais M, Mariette C, Batra SK, Aubert JP, Pigny P, Van Seuningen I (2004). A role for human MUC4 mucin gene, the ErbB2 ligand, as a target of TGF-beta in pancreatic carcinogenesis. Oncogene.

[8] Moniaux N, Chaturvedi P, Varshney GC, Meza JL, Rodriguez-Sierra JF, Aubert JP, Batra SK (2007). Human MUC4 mucin induces ultra-structural changes and tumorigenicity in pancreatic cancer cells. Br J Cancer.

[9] Moniaux N, Varshney GC, Chauhan SC, Copin MC, Jain M, Wittel UA, Andrianifahanana M, Aubert JP, Batra SK (2004). Generation and characterization of anti-MUC4 monoclonal antibodies reactive with normal and cancer cells in humans. J Histochem Cytochem.

[10] Ponnusamy MP, Singh AP, Jain M, Chakraborty S, Moniaux N, Batra SK (2008). MUC4 activates HER2 signalling and enhances the motility of human ovarian cancer cells. Br J Cancer.

[11] Singh AP, Chaturvedi P, Batra SK (2007). Emerging roles of MUC4 in cancer: a novel target for diagnosis and therapy. Cancer Res.

[12] Tsutsumida H, Goto M, Kitajima S, Kubota I, Hirotsu Y, Wakimoto J, Batra SK, Imai K, Yonezawa S (2007). MUC4 expression correlates with poor prognosis in small-sized lung adenocarcinoma. Lung Cancer.

[13] Tsutsumida H, Goto M, Kitajima S, Kubota I, Hirotsu Y, Yonezawa S (2004). Combined status of MUC1 mucin and surfactant apoprotein A expression can predict the outcome of patients with small-size lung adenocarcinoma. Histopathology.

[14] Yonezawa S, Goto M, Yamada N, Higashi M, Nomoto M (2008). Expression profiles of MUC1, MUC2, and MUC4 mucins in human neoplasms and their relationship with biological behavior. Proteomics.

[15] Yamada N, Kitamoto S, Yokoyama S, Hamada T, Goto M, Tsutsumida H, Higashi M, Yonezawa S (2011). Epigenetic regulation of mucin genes in human cancers. Clin Epigenetics.

[16] Yamada N, Nishida Y, Tsutsumida H, Goto M, Higashi M, Nomoto M, Yonezawa S (2009). Promoter CpG methylation in cancer cells contributes to the regulation of MUC4. Br J Cancer.

[17] Yamada N, Nishida Y, Tsutsumida H, Hamada T, Goto M, Higashi M, Nomoto M, Yonezawa S (2008). MUC1 expression is regulated by DNA methylation and histone H3 lysine 9 modification in cancer cells. Cancer Res.

[18] Yokoyama S, Kitamoto S, Higashi M, Goto Y, Hara T, Ikebe D, Yamaguchi T, Arisaka Y, Niihara T, Nishimata H, Tanaka S, Takaori K, Batra SK (2014). Diagnosis of pancreatic neoplasms using a novel method of DNA methylation analysis of mucin expression in pancreatic juice. PLoS One.

[19] Yokoyama S, Kitamoto S, Yamada N, Houjou I, Sugai T, Nakamura S, Arisaka Y, Takaori K, Higashi M, Yonezawa S (2012). The application of methylation specific electrophoresis (MSE) to DNA methylation analysis of the 5′ CpG island of mucin in cancer cells. BMC Cancer.

[20] Yokoyama S, Higashi M, Kitamoto S, Oeldorf M, Knippschild U, Kornmann M, Maemura K, Kurahara H, Wiest E, Hamada T, Kitazono I, Goto Y, Tasaki T (2016). Aberrant methylation of MUC1 and MUC4 promoters are potential prognostic biomarkers for pancreatic ductal adenocarcinomas. Oncotarget.

[21] Ehrlich M, Buchanan KL, Tsien F, Jiang G, Sun B, Uicker W, Weemaes CM, Smeets D, Sperling K, Belohradsky BH, Tommerup N, Misek DE, Rouillard JM (2001). DNA methyltransferase 3B mutations linked to the ICF syndrome cause dysregulation of lymphogenesis genes. Hum Mol Genet.

[22] Liang P, Song F, Ghosh S, Morien E, Qin M, Mahmood S, Fujiwara K, Igarashi J, Nagase H, Held WA (2011). Genome-wide survey reveals dynamic widespread tissue-specific changes in DNA methylation during development. BMC Genomics.

[23] Reik W (2007). Stability and flexibility of epigenetic gene regulation in mammalian development. Nature.

[24] Xu Y, Wu F, Tan L, Kong L, Xiong L, Deng J, Barbera AJ, Zheng L, Zhang H, Huang S, Min J, Nicholson T, Chen T (2011). Genome-wide regulation of 5hmC, 5mC, and gene expression by Tet1 hydroxylase in mouse embryonic stem cells. Mol Cell.

[25] Li E, Bestor TH, Jaenisch R (1992). Targeted mutation of the DNA methyltransferase gene results in embryonic lethality. Cell.

[26] Okano M, Bell DW, Haber DA, Li E (1999). DNA methyltransferases Dnmt3a and Dnmt3b are essential for de novo methylation and mammalian development. Cell.

[27] Ito S, Shen L, Dai Q, Wu SC, Collins LB, Swenberg JA, He C, Zhang Y (2011). Tet proteins can convert 5-methylcytosine to 5-formylcytosine and 5-carboxylcytosine. Science.

[28] Guo JU, Su Y, Zhong C, Ming GL, Song H (2011). Hydroxylation of 5-methylcytosine by TET1 promotes active DNA demethylation in the adult brain. Cell.

[29] Hackett JA, Zylicz JJ, Surani MA (2012). Parallel mechanisms of epigenetic reprogramming in the germline. Trends Genet.

[30] He YF, Li BZ, Li Z, Liu P, Wang Y, Tang Q, Ding J, Jia Y, Chen Z, Li L, Sun Y, Li X, Dai Q (2011). Tet-mediated formation of 5-carboxylcytosine and its excision by TDG in mammalian DNA. Science.

[31] Hitoshi S, Ishino Y, Kumar A, Jasmine S, Tanaka KF, Kondo T, Kato S, Hosoya T, Hotta Y, Ikenaka K (2011). Mammalian Gcm genes induce Hes5 expression by active DNA demethylation and induce neural stem cells. Nat Neurosci.

[32] Haffner MC, Chaux A, Meeker AK, Esopi DM, Gerber J, Pellakuru LG, Toubaji A, Argani P, Iacobuzio-Donahue C, Nelson WG, Netto GJ, De Marzo AM, Yegnasubramanian S (2011). Global 5-hydroxymethylcytosine content is significantly reduced in tissue stem/progenitor cell compartments and in human cancers. Oncotarget.

[33] Lian CG, Xu Y, Ceol C, Wu F, Larson A, Dresser K, Xu W, Tan L, Hu Y, Zhan Q, Lee CW, Hu D, Lian BQ (2012). Loss of 5-hydroxymethylcytosine is an epigenetic hallmark of melanoma. Cell.

[34] Xu W, Yang H, Liu Y, Yang Y, Wang P, Kim SH, Ito S, Yang C, Xiao MT, Liu LX, Jiang WQ, Liu J, Zhang JY (2011). Oncometabolite 2-hydroxyglutarate is a competitive inhibitor of alpha-ketoglutarate-dependent dioxygenases. Cancer Cell.

[35] Yang H, Liu Y, Bai F, Zhang JY, Ma SH, Liu J, Xu ZD, Zhu HG, Ling ZQ, Ye D, Guan KL, Xiong Y (2013). Tumor development is associated with decrease of TET gene expression and 5-methylcytosine hydroxylation. Oncogene.

[36] Yu M, Hon GC, Szulwach KE, Song CX, Zhang L, Kim A, Li X, Dai Q, Shen Y, Park B, Min JH, Jin P, Ren B (2012). Base-resolution analysis of 5-hydroxymethylcytosine in the mammalian genome. Cell.

[37] Ihaka R, Gentleman R. R (1996). A Language for Data Analysis and Graphics. Journal of Computational and Graphical Statistics.

