# Supplementary material for: TET1-mediated DNA hypomethylation regulates the expression of MUC4 in lung cancer
Source: Genes Cancer. 2017 Mar;8(3-4):517–27. doi: 10.18632/genesandcancer.139 (PMC5489649; doi:10.18632/genesandcancer.139)
Supplement: Supplementary file 1 [file ganc-08-517-s001.pdf]

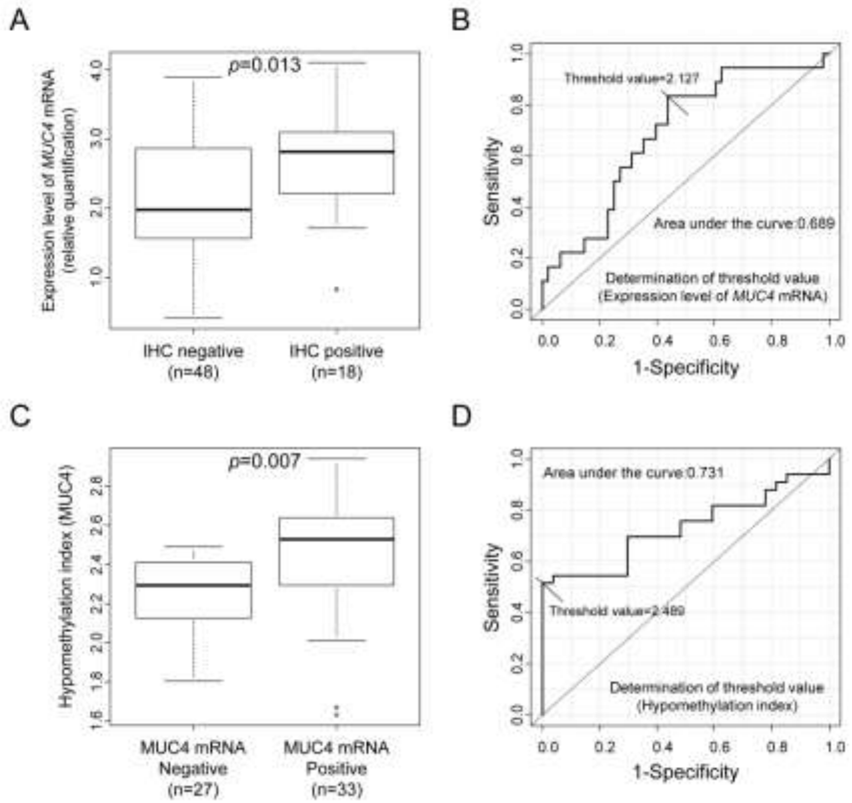

Supplementary Figure 1: ROC analysis for determination of threshold value. (A) Comparison of the expression level of *MUC4* mRNA between the group found to be MUC4 negative and the group found to be MUC4 positive by IHC analysis. The IHC positive group showed a higher expression level of *MUC4* than the IHC negative group. (B) Calculation of the threshold value of *MUC4* mRNA between MUC4 negative and MUC4 positive groups (as determined by IHC analysis). The threshold value (2.127) of mRNA expression between the MUC4 negative and MUC4 positive groups was calculated by ROC analysis. (C) Comparison of the hypomethylation index of the *MUC4* promoter between *MUC4* negative and *MUC4* positive groups (mRNA expression). The *MUC4* mRNA-positive group showed a higher hypomethylation index than the *MUC4* mRNA negative group. (D) Calculation of the threshold value of the *MUC4* hypomethylation index between *MUC4* negative and *MUC4* positive groups (mRNA expression). The threshold value (2.489) to differentiate between hypo-methylation and hyper-methylation of the *MUC4* promoter was calculated by ROC analysis.

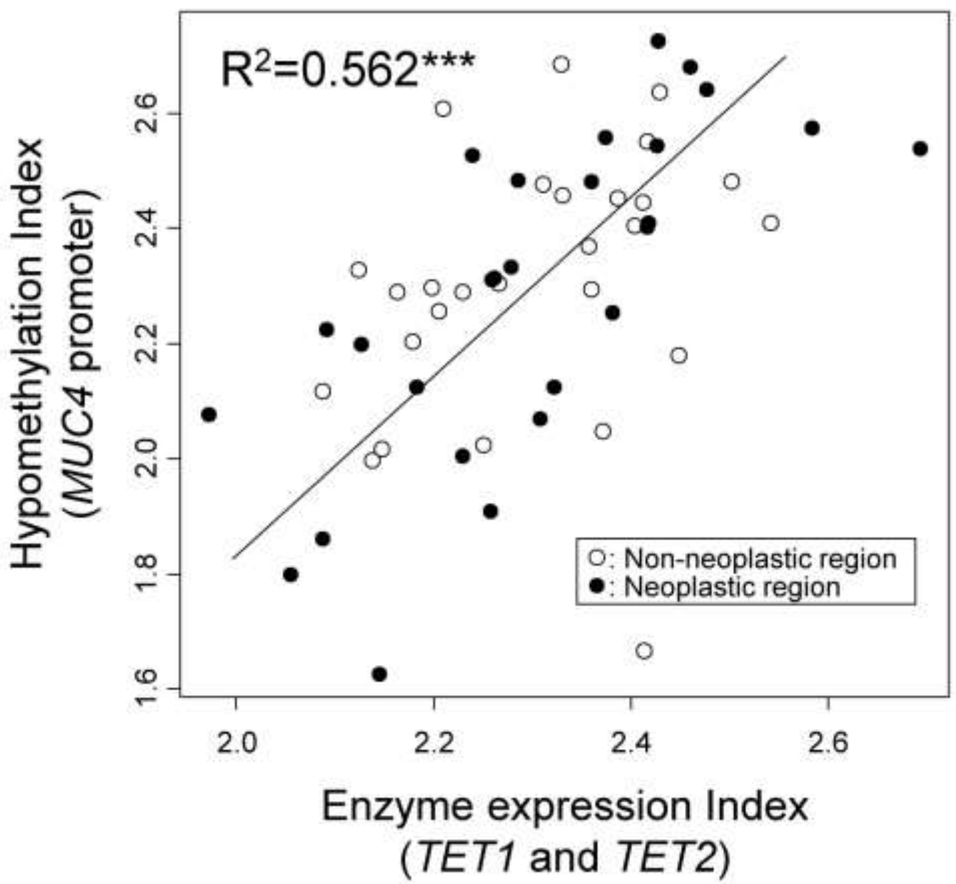

Supplementary Figure 2: Multiple regression analysis of the expression level of DNA demethylation-related enzymes against the hypomethylation status of the *MUC4* promoter. The multiple regression predictive value was obtained with the following formula: Fm (Enzyme expression index for *MUC4*) = 1.8 + 0.23(*TET1*) + 0.17(*TET2*). R2: R squared, ***: $p<0.001$, **: $p<0.01$, *: $p<0.05$, ○: non-neoplastic region, ●: neoplastic region.

Table S1. Clinicopathological features

Age	Median	±SD	
	69	±9.0	
Sex		n	(%)
	F	18	(54.5%)
	M	15	(45.4%)
Size	Median	±SD (mm)	
	25	±10.2	
T		n	(%)
	1a	8	(24.2%)
	1b	10	(30.3%)
	2a	12	(36.3%)
	2b	2	(6%)
	3	1	(3%)
N		n	(%)
	negative	31	(93.9%)
	positive	2	(6%)
M		n	(%)
	negative	32	(96.9%)
	positive	1	(3%)
ly		n	(%)
	negative	9	(27.2%)
	positive	24	(72.7%)
v		n	(%)
	negative	19	(57.5%)
	positive	14	(42.4%)
Pathology		n	(%)
	Adenocarcinoma		
	n=	31	(91.1%)
	Keratinizing squamous cell carcinoma		
	n=	2	(5.8%)
	Adenosquamous carcinoma		
	n=	1	(2.9%)
	Solid adenocarcinoma		
	n=	1	(2.9%)

Papillary adenocarcinoma			
	n=	25	(73.5%)
Lepidic adenocarcinoma			
	n=	17	(50%)
Mucinous adenocarcinoma			
	n=	4	(11.7%)
Micropapillary carcinoma			
	n=	3	(8.8%)

Table S2. Synthetic oligonucleotides used in the study

RT-PCR				
	MUC1		Forward:	5’-CCAGCACCGACTACTACCAAGAG-3’
			Reverse:	5’-CGTCGTGGACATTGATGGT-3’
	MUC4		Forward:	5’-TGGGACGATGCTGACTTCTC-3’
			Reverse:	5’-CCCCGTTGTTTGTCATCTTTC-3’
	TET1		Forward:	5'-CCCGAATCAAGCGGAAGAATA-3'
			Reverse:	5'-TACTTCAGGTTGCACGGT-3'
	TET2		Forward:	5'-AAGGCTGAGGGACGAGAACGA-3'
			Reverse:	5'-TGAGCCCATCTCCTGCTTCCA-3'
	TET3		Forward:	5'-CCTGCCGATGACAAGCTGGA-3'
			Reverse:	5'-GAGTTCCCGGATAGAGGCGA-3'
	AID		Forward:	5'-AAAATGTCCGCTGGGCTAAG-3'
			Reverse:	5'-AGGTCCCAGTCCGAGATGTAG-3'
	DNMT1		Forward:	5'-GAGGAAGCTGCTAAGGACTAGTTC-3'
			Reverse:	5'-ACTCCACAATTTGATCACTAAATC-3'
	DNMT3a		Forward:	5'-ACAAGAATGCCACCAAAGCAG-3'
			Reverse:	5'-TCATCCACCAAGACACAATGC-3'
	CAIX		Forward:	5'-CGGAAGAAAACAGTGCCTATGAG-3'
			Reverse:	5'-CAGGGCGGTGTAGTCAGAGA-3'
	β-actin		Forward:	5'-CTCTTCCAGCCTTCCTTCCTG-3'
			Reverse:	5'-GAAGCATTTGCGGTGGACGAT-3'
MSE				
	MUC1	1st	Forward:	5’-AAAGGGGGAGGTTAGTTGGA-3’
			Reverse:	5’-TACCCCTCACCTATAAACAC-3’
		2nd	Forward:	5’-[GC clamp*]AAGAGGTAGGAGGTAGGGGA-3’
			Reverse:	5’-AAAACAAAACAAATTCAAAC-3’
	MUC4	1st	Forward:	5’-AGAGTAAGGGGTGTATGGGTG-3’
			Reverse:	5’-AACCTACCCCCTTCATAAC-3’
		2nd	Forward:	5’-[GC clamp*]AGGAGAGAAAAGGGTGATTAG-3’
			Reverse:	5’-ACTCCACTACCCAACAACTAC-3’

*GC clamp: 5'-CGCCCGCCGCGCGCGGCGGGCGGGGCGGGGGCACGGGGGG-3'

Table S3. Comparison of expression level or methylation index between permeation negative and positive.

a) Non-neoplastic region

	Vascular permeation								Lymphatic permeation								Tumor size (TS)							
	negative			positive					negative			positive					TS < 20 mm			TS > 20mm				
	n	mean	±SD	n	mean	±SD	*p* value		n	mean	±SD	n	mean	±SD	*p* value		n	mean	±SD	n	mean	±SD	*p* value	
Methylation index of *MUC4* promoter																								
5mC	(19)	59.4	±22.5	(12)	70.7	±28.1	0.226		(8)	58.3	±27.3	(23)	65.8	±24.5	0.475		(6)	41.6	±18.9	(25)	69.1	±23.6	**0.013**	
5hmC	(19)	19.2	±21.0	(14)	17.8	±12.4	0.506	**	(9)	29.3	±28.0	(24)	14.6	±10.0	0.160	*	(8)	24.4	±28.3	(25)	16.8	±13.0	0.486	*
Expression level of mRNA																								
MUC4	(19)	2.25	±0.78	(14)	2.13	±0.72	0.666		(9)	2.05	±0.67	(24)	2.25	±0.78	0.509		(8)	2.23	±0.82	(25)	2.18	±0.74	0.876	
TET1	(19)	1.89	±0.44	(14)	1.79	±0.45	0.532		(9)	2.00	±0.51	(24)	1.79	±0.41	0.226		(8)	1.97	±0.45	(25)	1.81	±0.44	0.375	
TET2	(17)	0.28	±0.75	(14)	0.25	±0.66	0.895		(7)	0.18	±0.51	(24)	0.29	±0.76	0.728		(7)	-0.01	±0.66	(24)	0.35	±0.70	0.238	
TET3	(18)	2.70	±0.67	(14)	2.46	±0.77	0.355		(8)	2.34	±0.87	(24)	2.68	±0.66	0.258		(7)	2.21	±0.88	(25)	2.70	±0.65	0.113	
AICDA	(19)	2.40	±0.55	(14)	2.27	±0.56	0.490		(9)	2.52	±0.41	(24)	2.28	±0.58	0.268		(8)	2.53	±0.54	(25)	2.28	±0.55	0.273	
GCM1	(19)	1.70	±0.48	(14)	1.58	±0.33	0.415		(9)	1.83	±0.29	(24)	1.58	±0.45	0.121		(8)	1.91	±0.37	(25)	1.56	±0.41	**0.039**	
Dnmt1	(18)	0.01	±0.55	(14)	-0.06	±0.53	0.729		(9)	-0.09	±0.45	(23)	0.01	±0.57	0.647		(8)	0.00	±0.57	(24)	-0.03	±0.53	0.921	
Dnmt3a	(18)	0.09	±0.38	(14)	0.01	±0.40	0.575		(9)	0.27	±0.30	(23)	-0.03	±0.39	**0.041**		(8)	0.28	±0.33	(24)	-0.02	±0.38	0.060	

b) Neolpastic region

	Vascular permeation								Lymphatic permeation								Tumor size (TS)							
	negative			positive					negative			positive					TS < 20 mm			TS > 20mm				
	n	mean	±SD	n	mean	±SD	*p* value		n	mean	±SD	n	mean	±SD	*p* value		n	mean	±SD	n	mean	±SD	*p* value	
Methylation index of *MUC4* promoter																								
5mC	(19)	60.4	±27.5	(12)	72.8	±32.5	0.263		(8)	52.9	±30.4	(23)	69.5	±28.8	0.176		(6)	50.8	±30.0	(25)	68.6	±29.1	0.189	
5hmC	(19)	39.8	±40.9	(14)	20.8	±12.3	0.199	**	(9)	31.8	±24.8	(24)	31.7	±36.1	0.994		(8)	45.7	±39.7	(25)	27.3	±30.2	0.254	**
Expression level of mRNA																								
MUC4	(19)	2.44	±0.76	(14)	2.45	±1.07	0.962		(9)	2.58	±0.73	(24)	2.39	±0.95	0.599		(8)	2.70	±0.61	(25)	2.36	±0.95	0.351	
TET1	(19)	1.99	±0.64	(14)	1.83	±0.62	0.488		(9)	2.33	±0.76	(24)	1.77	±0.51	**0.020**		(8)	2.40	±0.69	(25)	1.77	±0.54	**0.011**	
TET2	(17)	0.34	±0.51	(14)	0.22	±0.65	0.588		(7)	0.46	±0.46	(24)	0.24	±0.60	0.375		(7)	0.50	±0.40	(24)	0.22	±0.61	0.264	
TET3	(18)	2.45	±0.93	(14)	2.72	±0.58	0.346		(8)	2.49	±0.71	(24)	2.59	±0.83	0.780		(7)	2.55	±0.71	(25)	2.57	±0.83	0.955	
AICDA	(19)	2.32	±0.60	(14)	2.28	±0.72	0.868		(9)	2.58	±0.63	(24)	2.20	±0.62	0.127		(8)	2.43	±0.58	(25)	2.27	±0.67	0.541	
GCM1	(19)	1.63	±0.68	(14)	1.58	±0.62	0.815		(9)	1.89	±0.76	(24)	1.51	±0.58	0.130		(8)	1.82	±0.63	(25)	1.54	±0.65	0.295	
Dnmt1	(18)	0.08	±0.64	(14)	-0.05	±0.52	0.527		(9)	0.37	±0.57	(23)	-0.11	±0.54	**0.032**		(8)	0.31	±0.47	(24)	-0.07	±0.60	0.117	
Dnmt3a	(18)	0.11	±0.46	(14)	-0.06	±0.49	0.330		(9)	0.40	±0.45	(23)	-0.10	±0.41	**0.005**		(8)	0.38	±0.43	(24)	-0.08	±0.44	**0.014**	

c) comparison between neoplasm and non-neoasm

	Vascular permeation positive							Lymphatic permeation positive								Early stage (TS < 20 mm)						
	non-neoplastic region			neoplastic region				non-neoplastic region			neoplastic region					non-neoplastic region			neoplastic region			
	n	mean	±SD	n	mean	±SD	*p* value [p]	n	mean	±SD	n	mean	±SD	*p* value [p]		n	mean	±SD	n	mean	±SD	*p* value [p]
Methylation index of *MUC4* promoter																						
5mC	(12)	70.7	±28.1	(12)	72.8	±32.5	0.707	(23)	65.8	±24.5	(23)	69.5	±28.8	0.370		(6)	41.6	±18.9	(6)	50.8	±30.0	0.257
5hmC	(14)	17.8	±12.4	(14)	20.8	±12.3	0.420	(24)	14.6	±10.0	(24)	31.7	±36.1	**0.004**	**	(8)	24.4	±28.3	(8)	45.7	±39.7	0.297
Expression level of mRNA																						
MUC4	(14)	2.13	±0.72	(14)	2.45	±1.07	0.178	(24)	2.25	±0.78	(24)	2.39	±0.95	0.384		(8)	2.23	±0.82	(8)	2.70	±0.61	0.084
TET1	(14)	1.79	±0.45	(14)	1.83	±0.62	0.770	(24)	1.79	±0.41	(24)	1.77	±0.51	0.750		(8)	1.97	±0.45	(8)	2.40	±0.69	**0.009**
TET2	(14)	0.25	±0.66	(14)	0.22	±0.65	0.861	(24)	0.29	±0.76	(24)	0.24	±0.60	0.579		(7)	-0.01	±0.66	(7)	0.50	±0.40	**0.016**
TET3	(14)	2.46	±0.77	(14)	2.72	±0.58	0.157	(24)	2.68	±0.66	(24)	2.59	±0.83	0.563		(7)	2.21	±0.88	(7)	2.55	±0.71	0.500
AICDA	(14)	2.27	±0.56	(14)	2.28	±0.72	0.901	(24)	2.28	±0.58	(24)	2.20	±0.62	0.512		(8)	2.53	±0.54	(8)	2.43	±0.58	0.567
GCM1	(14)	1.58	±0.33	(14)	1.58	±0.62	0.987	(24)	1.58	±0.45	(24)	1.51	±0.58	0.485		(8)	1.91	±0.37	(8)	1.82	±0.63	0.679
Dnmt1	(14)	-0.06	±0.53	(14)	-0.05	±0.52	0.934	(23)	0.01	±0.57	(23)	-0.11	±0.54	0.213		(8)	0.00	±0.57	(8)	0.31	±0.47	0.179
Dnmt3a	(14)	0.01	±0.40	(14)	-0.06	±0.49	0.357	(23)	-0.03	±0.39	(23)	-0.10	±0.41	0.089		(8)	0.28	±0.33	(8)	0.38	±0.43	0.194

ks.test is Kolmogorov–Smirnov test. Performs an F test to compare the variances of two samples from normal populations. p value is calculated by equal variance t test, Unequal variance t test (*) or Mann-Whitney U test (**), p value p is calculated by paired t test. n: negative, p: positive, N: non-neoplastic region, T: neoplastic region, E: Early stage (T score=1a), L: Late stage (T score>1a)
